# Developmental expression of DAX1 in the European sea bass, Dicentrarchus labrax: lack of evidence for sexual dimorphism during sex differentiation

**DOI:** 10.1186/1477-7827-5-19

**Published:** 2007-05-30

**Authors:** Rute ST Martins, Laurence AM Deloffre, Constantinos C Mylonas, Deborah M Power, Adelino VM Canário

**Affiliations:** 1 Centro de Ciências do Mar, CIMAR-Laboratório Associado, University of Algarve, Faro, Portugal; 2 Hellenic Centre for Marine Research, Institute of Aquaculture, P.O. Box 2214, Iraklion, Crete 71003, Greece

## Abstract

**Background:**

DAX1 (NR0B1), a member of the nuclear receptors super family, has been shown to be involved in the genetic sex determination and in gonadal differentiation in several vertebrate species. In the aquaculture fish European sea bass, Dicentrarchus labrax, and in the generality of fish species, the mechanisms of sex determination and differentiation have not been elucidated. The present study aimed at characterizing the European DAX1 gene and its developmental expression at the mRNA level.

**Methods:**

A full length European sea bass DAX1 cDNA (sbDAX1) was isolated by screening a testis cDNA library. The structure of the DAX1 gene was determined by PCR and Southern blot. Multisequence alignments and phylogenetic analysis were used to compare the translated sbDAX1 product to that of other vertebrates. sbDAX1 expression was analysed by Northern blot and relative RT-PCR in adult tissues. Developmental expression of mRNA levels was analysed in groups of larvae grown either at 15°C or 20°C (masculinising temperature) during the first 60 days, or two groups of fish selected for fast (mostly females) and slow growth.

**Results:**

The sbDAX1 is expressed as a single transcript in testis and ovary encoding a predicted protein of 301 amino acids. A polyglutamine stretch of variable length in different DAX1 proteins is present in the DNA binding domain. The sbDAX1 gene is composed of two exons, separated by a single 283 bp intron with conserved splice sites in same region of the ligand binding domain as other DAX1 genes. sbDAX1 mRNA is not restricted to the brain-pituitary-gonadal axis and is also detected in the gut, heart, gills, muscle and kidney. sbDAX1 mRNA was detected as early as 4 days post hatching (dph) and expression was not affected by incubation temperature. Throughout gonadal sex differentiation (60–300 dph) no dimorphic pattern of expression was observed.

**Conclusion:**

The sbDAX1 gene and putative protein coding region is highly conserved and has a wide pattern of tissue expression. Although gene expression data suggests sbDAX1 to be important for the development and differentiation of the gonads, it is apparently not sex specific.

## Background

*DAX1 *(*NR0B1*) is a member of the nuclear receptors super family initially isolated from a 160 kb region in the human Xp21 chromosome, called *dosage- sensitive sex reversal (DSS) *region, that, when duplicated in the genome, is responsible for the *DSS *syndrome which causes male to female sex reversal in individuals with a normal *SRY *gene [1]. Mutations in this gene can give rise to a severe adrenal insufficiency, adrenal hypoplasia congenita frequently accompanied by hypogonadotropic hypogonadism [2]. Similarly, over expression of the DAX1 gene in certain mouse strains also leads to phenotypic sex reversal in males [3]. Mice knockout models have also shown that DAX1 expression in developing testicular tissue is essential for normal testicular development and for male fertility. DAX1 deficient male mice present abnormal testicular morphology, low sperm production and sperm count [4,5], whereas in female DAX1 knockout mice, normal ovarian development and reproductive function is accomplished, although presenting a compromised endocrine function.

DAX1 is unusual in that it lacks the characteristic zinc-finger motif in the DNA-binding domain that is highly conserved in other members of the family [2]. In humans it encodes a protein of 470 amino acids, which N-terminus consists instead of three repeats of a unique 67 amino acids long cysteine-rich motif [2]. The number of repeats seems to vary throughout evolution, since in all homolog of non mammalian species studied so far only one repeat is present: chicken [6] alligator [7], frog [8] and in Nile tilapia [9]. In the C-terminus, there is a nuclear receptor ligand-binding domain motif, even though no ligands are known to bind the protein [10], and a bipartite transcriptional repression domain [11]. DAX1 has been shown to be able to down regulate the transcription of several genes involved in the development and steroidogenic activity of both the adrenal and gonadal tissues, e.g. *StAR *[11], *CYP11A *[12], *CYP17 *[13], *CYP19 *[14], müllerian inhibiting substance (*MIS*) [15], estrogens receptors α and β [16] and androgen receptor [17]. Steroidogenic factor 1 (SF-1), another nuclear receptor, also regulates most of the same steroidogenic enzymes which DAX1 targets and it is the balance between repressor and inducer function of DAX-1 and SF-1 that is critical for the regulation of these enzymes [18]. Transcriptional repression by DAX1 is thought to be mediated by both direct binding to gene promoters, e.g *StAR *[11], and by interaction with different co-repressors: N-CoR [19] and Alien [20]. In addition, DAX1 is also suggested to play additional regulatory function in the post-transcriptional processes [18].

The European sea bass, *Dicentrarchus labrax*, is a gonochoristic teleost fish belonging to the family *Moronidae*, closely related to the Serranidae, a family composed of hermaphroditic species [21]. This singularity has been suggested as being connected to its sex liability translated in the occurrence of intratesticular oocytes in juveniles [21] and to a high degree of temperature-dependent sex differentiation (TSD) in farming conditions [22-25]. Thus, incubating sea bass larvae at relatively high temperatures (above 17°C) strongly affects the sex ratio, increasing the proportion of males [22-25]. The thermosensitive period for gonadal differentiation in sea bass corresponds to the first 60 days post fertilization (dpf) [23]. The gonads become sensitive to steroids during a period of around 6 weeks centred at 100 dpf [21]. The first gonad to differentiate is the ovary at 160 dpf (8 cm standard length, SL) followed by the testis, most males differentiating at 250 dpf (11.8 cm SL) [21]. Size is a critical marker for the timing of sexual differentiation, so that larger larvae are more likely to develop as females while smaller larvae tend to develop as males [26]. Thus, the identification of genes acting early in the sex determining cascade will facilitate understanding the mechanism involved in the TSD in this species and may provide markers for the early detection of sex.

In teleost fish, including the sea bass, exogenous steroids given at the appropriate period of sex differentiation can lead to various degrees of sex change including sex reversal [27,28]. Thus natural and artificial estrogens feminize the gonads while non-aromatizable androgens and aromatase inhibitors have a masculinising effect [27,28]. Furthermore, downregulation of *CYP19 *transcripts has been associated to temperature-induced masculinisation in Nile tilapia [29,30] as well as with testis differentiation in reptiles with TSD [31].

**Figure 1:**
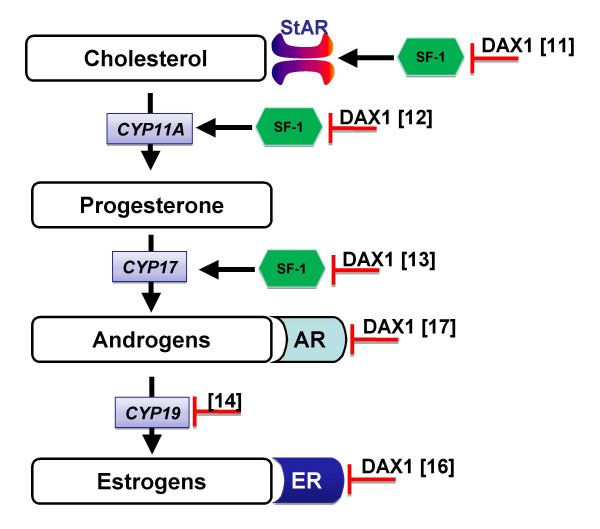
**Schematic representation of pathway for the biosynthesis of sexual steroids**. DAX1 regulates key steps for androgen production by repressing the transcriptional activation of SF-1 on the promoters of *StAR *[11], *CYP11A *[12] and *CYP17 *[13]. DAX1 binds directly to the *CYP19 *promoter to downregulate its transcription [14]. It also regulates estrogen [16] and androgen receptors [17] through protein-protein interactions. *CYP11A*, *CYP17 *and *CYP19 *are, respectively, genes that encode for the P450 enzymes cholesterol side chain cleavage, 17-hydroxylase and aromatase. StAR is the mitochondrial cholesterol transporter protein. AR and ER are respectively androgen and estrogen receptors (α and β). Supporting references for the scheme are indicated by numbers within [].

Considering the above, and the high conservation of the main features of the sex differentiation pathway in vertebrates [7], we hypothesize that in sea bass DAX1 expression may be directly or indirectly upregulated by high temperatures and, through the interaction of sex steroid producing enzymes or receptors, promote the masculinisation of the gonads (Figure 1). In this report we investigate the possible role of DAX1 on sex differentiation in the European sea bass. We have cloned and characterized a cDNA for a European sea bass DAX1- homolog, determined the gene structure and analysed its mRNA expression pattern in early gonadal development in fish cultured at normal and masculinising temperatures and during gonadal differentiation in female and male biased populations.

## Methods

### Animals and experiments

Juvenile and adult European sea bass used to provide RNA for cloning and gene expression analysis in different tissues were obtained from Timar, Cultura de Águas (Livramento, Portugal) and maintained at the Ramalhete Experimental Station (University of Algarve, Faro, Portugal) prior to sampling in through-flow seawater tanks at 17 ± 2°C under natural photoperiod.

To investigate DAX1 gene expression during the thermosensitive period an experiment was carried out in which sea bass eggs were incubated at 15°C or 20°C until they reached a total length of 18 mm (48 and 66 dph, respectively). Thereafter both groups were maintained at natural temperature (ranging between 18 to 22°C) until sex determination was complete (300 dph) and gonadal sex was determined histologically. Rearing conditions were similar to those described by Mylonas et al [22]. Treatment resulted in 75% and 22% females in the 15°C group and 20°C groups respectively. Samples for gene expression analysis were taken at 4, 18, 32, 46 and 60 dph to analyze gene expression. At 4 dph samples were 6 pools of 3 larvae per sample, at other times consisted of 6 individual larvae.

The fish to analyse gene expression during gonadal development in male and female dominant populations came from an experiment described in detail by Papadaki et al. [32], carried out at the Institute of Aquaculture, Hellenic Center for Marine Research (Crete, Greece). Eggs were incubated at 17°C until hatching and from then on the larvae and juveniles were cultured at ambient temperatures throughout. The experiment consisted of grading the fish four times at ca. 50 days intervals, from 56 dph to 220 dph, each time retaining the 50% larger and the 50% smaller fish in the two groups that were formed after the first grading. Since European sea bass females tend to be larger than males, there was a progressive selection of females in the group with the larger fish and of males in the group with the smaller fish. Samples were taken from each grading but only from 150 days (second grading) it was possible to determine sex by histology. The percentage of females in the smaller fish population decreased from 50% as a result of the 120 days grading, 43% after the 166 days grading and 30.2% following 200 days. In the larger population during the same period the % females were 91%, 92% and 96.5%. Samples (n = 3–8 per point) to analyze gene expression were taken at 56 dph (whole larvae), at 100, 150, 200, 250 dph (body trunks) and at 300 dph (gonad).

For sample collection, fish were killed with an overdose of 2-phenoxyethanol (1:5000, Sigma-Aldrich, Madrid, Spain). Whole larvae or dissected tissues in older fish were immediately frozen in liquid nitrogen and stored at -80°C until used (normally within 3 months).

### RNA isolation and reverse transcription

Total RNA was isolated from larvae, gonads and other various tissues of adult sea bass (1.5 years old) with TRI reagent (Sigma-Aldrich, Madrid, Spain) according to the manufacturer's instructions. Total RNA (3–5 μg) was reverse-transcribed in 40 μl at 37°C for 60 min., using 200 units of superscript, 0.25 mM dNTPs, 0.2 units of RNase inhibitor and 3 μg random-hexamers. Poly (A)+ RNA was isolated from total RNA using a mRNA purification kit from Amersham Pharmacia Biosciences (Lisbon, Portugal).

### Isolation of the sea bass DAX1 full length cDNA

A pair of oligonucleotide primers (Tildax, Table 1) was designed based on the sequence of tilapia DAX1 [9] and used in reverse transcriptase-polymerase chain reaction (RT-PCR) to isolate a 587bp fragment of DAX1 from testis cDNA. RT-PCR amplification was carried out in 25 μl reactions containing 1 μl of cDNA, 2.5 μl of 10× Buffer (Promega), 2.0 mM MgCl_2_, 0.04 dNTPs, 0.4 μM sense and antisense primers, and 0.5 units of taq Polimerase (Promega) (see also Table 1). Products were separated and purified from an agarose gel (1%) with the GFX PCR DNA and Gel Purification Kit (Amersham Biosciences), cloned in pGem Teasy vector (Promega, Madison, USA) and sequenced. The nucleotide sequence of the PCR product had 90% similarity with that of tilapia DAX1 cDNA. The sea bass DAX1 fragment was radiolabeled using a random labelling kit (Rediprime II, Amersham-Pharmacia) and used to screen a sea bass testis cDNA library constructed in Lambda Zap using the UNIZAP vector (Stratagene). After screening 4 × 10^5 ^recombinants at moderate stringency (in Church- Gilbert buffer at 55°C), four independent clones were autoexcised in pBluescript phagemid using helper phage. Two clones, 2LD and 5LD, each containing a 1373 bp insert, were isolated and completely sequenced by primer walking. The two clones were identical.

**Table 1:** Primers and RT-PCR conditions for DAX1 isolation and gene expression analysis

Primer	Primer sequences (5'-3')	Thermocycles	Amplicon (bp)
TilDax1	CTGGTGAAGACGGTGCGGTTCGT	35 cycles:	587
	CACTATGGACCCGTGACCTACTT	94°C 1 min	
		57°C 1 min	
		72°C 1 min	
sbDax1	CTGGGGGGTTCTGGTGAAG	32 cycles:	161
	CGGTCTCCGTGGTCTCAAAGTC	94°C 1 min	
		59°C 1 min	
		72°C 1 min	
18S	TCAAGAACGAAAGTCGGAGG	18 cycles:	450
	GGACATCTAAGGGCATCACA	94°C 1 min	
		59°C 1 min	
		72°C 1 min	
F1	CTGGCGAAAACTTTCCGATTCCT		
F2	CTCGGCGGTTCTGGTGAAG		
F3	GTGGAGCCCAGCATGCTGCAGC		
R1	CGGTCTCCGTGGTCTCAAAGTC		
R2	AGCCTCCCTGTGAAGTGTCTGG		
R3	CTTCCCGTCACCGGTCTGAAGAAG		
R4	GCCAATGACCGGTTTGAAGAAG		

Primers Ti1Dax1, sbDAX1 and 18S were used to amplify cDNA in relative semi-quantitative RT-PCR. Primers F1-F3 sense primers were used in various combinations with antisense primers R1-R4 in the determination of gene structure.

### Multisequence alignment and phylogenetic analysis

The DAX1 sequences used in the alignments were from was mapped to Fugu Consortium Scaffold S000386 [33], tilapia [9], chicken [6], frog [8], human [2] and alligator [7]. Alignments were performed using the ClustalW [34]. Phylogenetic analysis was performed using the ProML -Protein Maximum Likelihood program (^© ^Copyright 1986–2001 by the University of Washington).

### Southern and northern blot analysis

High molecular weight DNA was isolated from liver of 3 females and 3 males, using a standard extraction method with phenol:chloroform. Ten μg were digested with 45 U ECORI (Takara Biomedicals, Shiga, Japan) at 37°C over night and the fragmented DNA was separated on agarose gel (0.7%), the gel was treated with a 0.250 M HCl, denatured in 1.5 M Nacl/0.5 M NaOH and neutralized in 1.5 M NaCl/0.5 M Tris Base, pH = 8. The DNA was transferred in 20 × SSC by capillarity onto a nylon membrane (Hybond XL, Amersham Pharmacia). The membrane was hybridized with (α-^32^P) CTP labelled 587 bp DAX1 cDNA fragment in Perfect Hyb solution (Sigma), according to the manufacturer's instructions. Probe labelling was by random priming (Rediprime II, Amersham-Pharmacia).

For northern blot, total mRNA from 3 testes and 3 ovaries was isolated from 2.5 years old fish as previously described. The RNA (5 μg for testis and 10 μg from females) was heat denatured for 5 min (65°C), run on a 1% denaturing formaldehyde gel, and transferred onto a nylon membrane (Hybond XL, Amersham Pharmacia). Hybridization with a DAX1 probe covering the full ORF (Open reading frame) followed the same procedure as described for Southern blotting analysis.

### Gene structure

Different sets of forward (F1-F3) and reverse (R1-R4) primers were designed according to the sbDAX1 full length sequence, and used in all possible combinations, to amplify the intronic sequences present within the DAX1 gene from liver genomic DNA, isolated as described.

### Relative RT-PCR analysis

In all RT-PCR reactions, 1 μl of cDNA was amplified in a reaction of 25 μl containing 2.5 μl of 10× Buffer (Promega), 2.0 mM MgCl_2_, 0.04 dNTPs, 0.4 μM of DAX1 specific primers (sbDAX1 Table 1), and 0.5 units of Taq polymerase (Promega). The thermocycles were optimised in order to obtain a single product within the linear range of RT-PCR amplification. For all samples RT-PCR amplification of 18S rRNA was used as reference in the same conditions as above except for 1.0 mM MgCl_2 _and 0.4 μM 18S primers (18S Fw/18S Rv, Table 1). The DAX1 and 18S RT-PCR reaction products were separated on agarose gel (0.7%), denatured (1.5 M Nacl/0.5 M NaOH), neutralized (1.5 M NaCl/0.5 M Tris Base, pH = 8) and transferred onto nylon membranes (Hybond XL, Amersham Pharmacia) in 6× SSC solution by a standard capillarity method. The membranes were hybridized with (α-^32^P) CTP radiolabeled probes (161 bp DAX1 and 450 bp 18S) in Church Gilbert buffer (65°C), and subjected to stringent washing (1 × SSC/0.1%SDS and 0.1 × SSC/0.1%SDS) at 65°C. The membrane signals were quantified by phosphorimaging (GS-505 Molecular Imager System, Biorad) and expressed as the ratio of amplified target to that of 18 S ribosomal RNA.

### Statistics

The ratios of DAX1 to 18S were log transformed and the effects of temperature or grading and age were analysed by two-way Analysis of Variance followed by the Tukey multiple comparison test with a significance level of 5%. Unless otherwise stated the results are expressed as mean ± standard error of the mean.

## Results

### Cloning of sea bass DAX1 and sequence analysis

**Figure 2:**
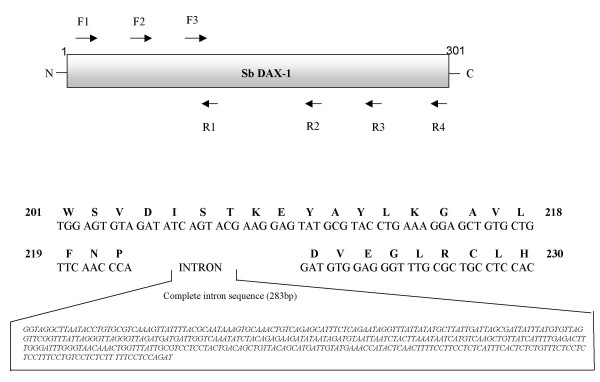
**The European sea bass DAX1 gene structure**. Different sets of forward (F1-F3) and reverse (R1-R4) primers were used to amplify the DAX1 gene from genomic DNA. PCR amplification yielded a single 381 bp intron sequence, positioned between amino acids 221 and 222 within the LBD.

A 1373 bp DAX1 clone (named sbDAX1) was obtained from screening approximately 4 × 10^5 ^recombinants of a testis cDNA library. The 5' untranslated region (UTR) of 116 bp ends with a partially conserved Kozak sequence (CCATGGC) signalling the beginning of an open reading frame (ORF) encoding 301 amino acids (additional file 1). The 3'UTR is approximately 350 bp, and contains a putative poly adenylation signal at nucleotides 1334–1340 and a poly(A)^+ ^tail. The sequence has been deposited to Embl (accession number AJ633646). PCR of sea bass genomic DNA resulted in the amplification of several DNA fragments covering the coding region of sbDAX1, but only the primer combination F3-R4 contained a 283 bp intron, located at amino acid positions 221–222, within the LBD (Figure 2). This intron is located in the exact position as all other DAX1 genes described so far, confirming that the structure of this gene is maintained throughout all taxa.

**Figure 3:**
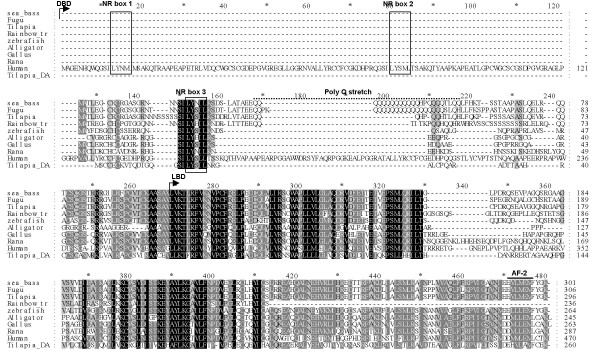
**Multisequence alignment of DAX1 protein**. Identical and similar amino acids are marked with asterisks and dots, respectively. The putative DNA-binding domain (DBD) and ligand binding domain (LBD) are indicated. The conserved LXXLL-like motif and the AF-2 core, in the DBD and LBD, respectively, are indicated inside boxes.

**Figure 4:**
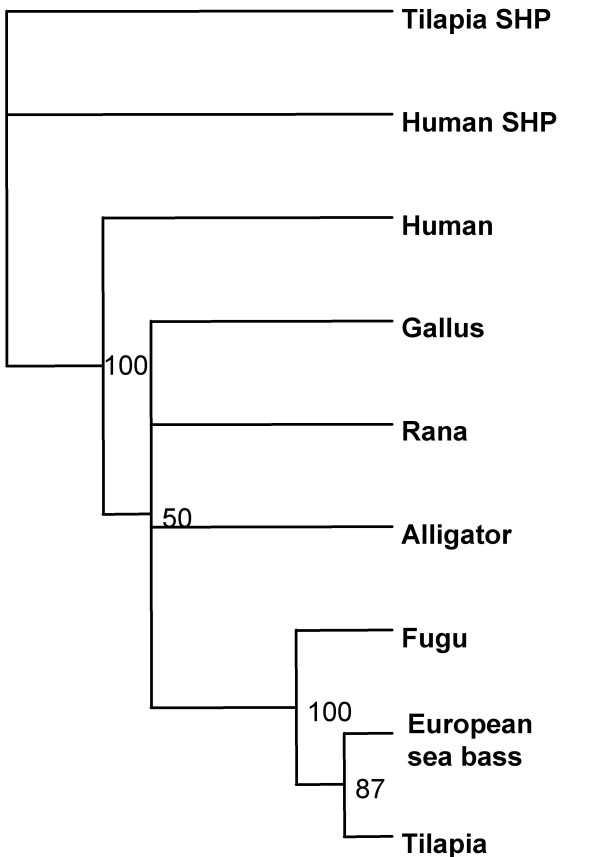
**Phylogenetic tree of DAX1 protein**. Consensus tree obtained by parsimony analysis with corresponding bootstrap values from sampling 1000 trees. Human (accession Q15466) and tilapia (accession AAN17674) small heterodimer partner (SHP) were used to root the tree. The other amino acid sequences used are mentioned in the Materials and Methods.

Multisequence alignment (Figure 3) revealed that the predicted sbDAX1 protein shared highest overall sequence identity and similarity to Fugu followed by, in descending order, tilapia, zebrafish, rainbow trout, chicken, frog, alligator and human (Table 2). While a high level of conservation was found for the carboxy terminal region (higher than 50%), the amino terminus was relatively more poorly conserved. The main reasons for the poor conservation of the putative DNA binding domain is the much longer N-terminal human protein compared to all vertebrates and the presence of region of variable length and amino acid sequence adjacent to a generally conserved nuclear LXXLL-like motif (NR box3, Figure 3). In the sea bass DAX1 this region corresponds to a poly glutamine stretch composed of 13 Glu (amino acid positions 36–48) and two extra glutamine repeats composed of 4 and 2 Glu (amino acid positions 51–54, 57–58). As with all the non-mammalian vertebrates the sbDAX1 protein lacked NR box1 and NR box2 of the three LXXLL-like repeats present in the human DAX1 DNA binding domain. Similarly, sea bass, like chicken, frog, alligator, tilapia, and fugu has only a partially conserved copy of NR box3 (-NSILYNILKS-) at amino acids positions 17–27 (Figure 3). Phylogenetic analysis shows that fish DAX1 cluster together with other fish DAX1 and separate from the tetrapods (Figure 4).

**Table 2:** Comparison of DAX1 protein sequence identity and similarity between European sea bass and other vertebrates.

Species	N-Terminal (%)	C-Terminal (%)	DAX1 protein (%)
Fugu	80 (89)	90 (94)	86 (92)
Tilapia	69 (78)	94 (96)	85 (89)
Frog	33 (44)	56 (73)	48 (62)
Alligator	19 (26)	59 (73)	43 (54)
Chicken	32 (39)	62 (78)	51 (62)
Human	11 (18)	49 (66)	25 (34)

Sequence identity and similarity (parentheses) of the DAX1 DNA binding domain (N-terminal), carboxy-terminal region (C-terminal) and whole protein (DAX1 protein) from different vertebrates (Species).

**Figure 5:**
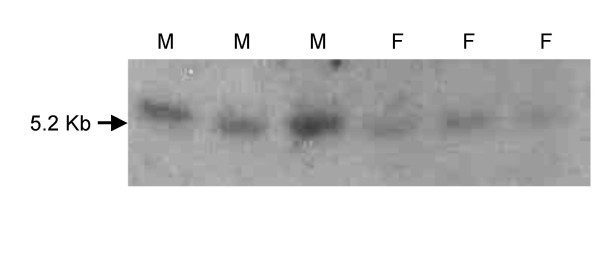
**Southern blot hybridizations of DAX1**. Genomic DNA of three males (M) and three females (F) was digested with EcoRI and hybridized with the sea bass DAX-1 gene.

**Figure 6:**
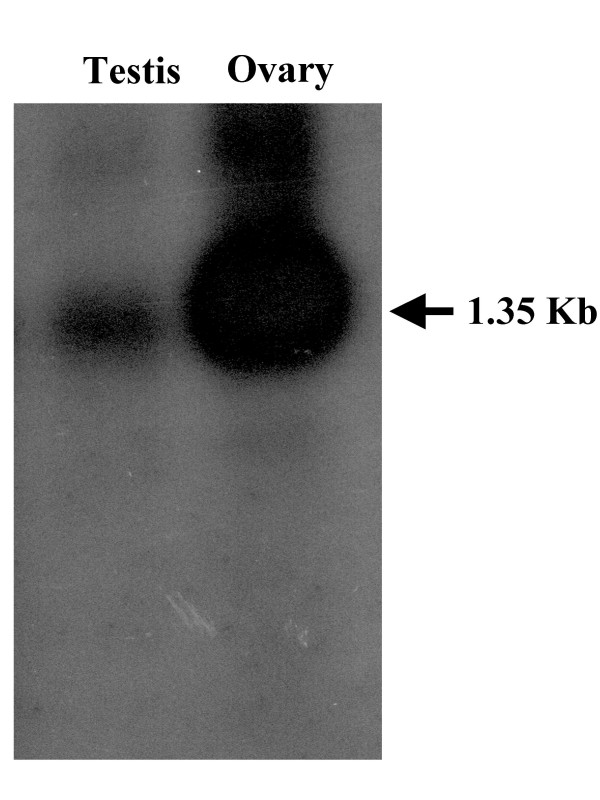
**Northern blot hybridizations of DAX1**. Total RNA from testis (5 μg) or ovary (10 μg) was run on 1% denaturing formaldehyde gel, transferred onto a nylon membrane and hybridized with the sea bass DAX1 full length cDNA.

Southern blot analysis showed a single band of similar size in DNA from both sexes (Figure 5). Northern blot analysis yielded a single transcript of ~1.3 kb in both ovary and testis (Figure 6).

### Tissue distribution of DAX1 mRNA expression

**Figure 7:**
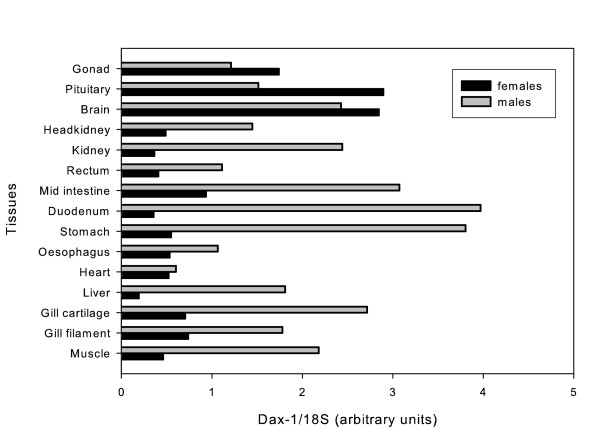
**DAX1 gene expression in different tissues from adult European sea bass**. RT-PCR of DAX1 relative to 18S in pools of tissues from three females or three males.

The pattern of sbDAX1 expression in female and male tissues was determined by relative RT-PCR analysis (Figure 7). sbDAX1 is widely expressed with a relatively balance expression in the two sexes in brain, pituitary and gonads, but an apparent male dominance in a range of other tissues, including head and trunk kidney, digestive tract, heart, liver, muscle and gills.

### DAX1 mRNA expression during early development

**Figure 8:**
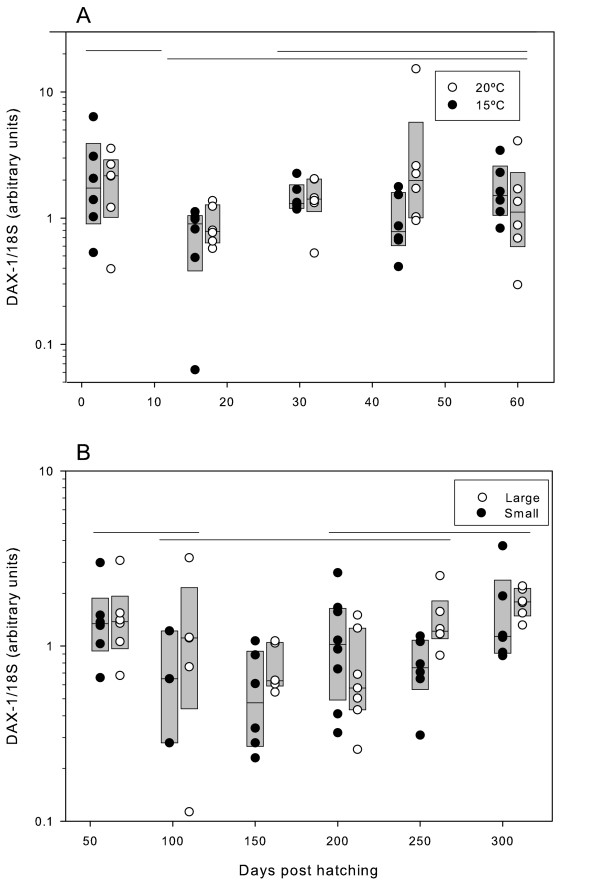
**DAX1 gene expression during early development in European sea bass**. RT-PCR of DAX1 relative to 18S in sea bass larvae. A) Whole larvae during the thermo sensitive period for sex determination (n = 6 at each sampling point). Larvae were grown at either 15°C or 20°C resulting, respectively, in 77.5% and 28% females. B) Whole larvae (56 days), body trunks (98, 150, 200 and 250 days) or gonads (300 days) during the period of sex differentiation (n = 3–8). Larvae were repeatedly graded according to size. Larger fish developed into a majority of females (96.0% females) and smaller fish developed into a majority of males (35.4% females). Groups covered by horizontal line at the same level are not statistically different.

The expression pattern of sbDAX1 during the temperature sensitive period for sex differentiation was determined by relative RT-PCR in larvae from 4 dph to 60 dph (Figure 8A). At 15°C sbDAX1 expression was always detected and at similar levels in larvae grown at 15°C and 21°C. The levels of sbDAX1 expression were significantly lower at 18 days, compared to any of the other points sampled (p < 0.05).

The expression pattern of sbDAX1 between 56 dph and 300 dph, encompassing gonadal differentiation, was determined by relative RT-PCR (Figure 8B). sbDAX1 expression was detected in all samples, and no significant differences were found between larger and smaller fish. In both groups significantly lower DAX1 expression was detected at 150 days (p < 0.005).

## Discussion

In this study we have identified a full length cDNA clone of a DAX1 homolog gene in the European sea bass. The sbDAX1 cDNA clone encodes a 301 amino acids protein that shared highest overall sequence identity and similarity to other DAX1 homologs in fish (fugu, tilapia, zebrafish and rainbow trout) followed by birds, reptiles and mammalians. As with all DAX1 genes described so far, the sea bass DAX1 gene lacks the characteristic zinc-finger motif in the DNA-binding domain (DBD) that is highly conserved in other nuclear receptors [2] but, as in other non mammalian species, teleost fish [Nile tilapia, 9], amphibians [frog, 8], reptiles [alligator, 7], and birds [chicken, 6], only one of the three LXXLL-like motifs present in the DNA-binding domain of the human DAX1 aligns at the same positions of the putative DBD (NR box 2 in Figure 3). It has been documented that these LXXLL-like motifs are implicated in nuclear receptor binding, namely in interacting with SF-1 [35,36], estrogen receptor [16], androgen receptor [17] and progestin receptor [37]. Moreover it has been shown that all LXXLL-like motifs in DAX1 are able to interact with other nuclear receptors, although with different binding affinities [16]. Thus it appears that DAX1 from sea bass and from other non mammalian vertebrates is able to interact with nuclear receptors through the conserved NR box 2.

Intriguingly, however, the sea bass and fugu first LXXLL-like motif is followed by a polyglutamine (polyQ) stretch. In sea bass, the polyQ stretch is composed of 13 glutamines followed by two extra glutamine repeats composed of 4 and 2 glutamines. In the fugu DAX1 the polyQ strech is also composed of 17 glutamines but it is uninterrupted. In contrast, Nile tilapia contains only seven scattered glutamines within this region, rainbow trout 6 and zebrafish only 3 [38]. Glutamine is the most common repeated amino acid in eukaryotic proteins, and it has been postulated that the introduction of such repeats is a general evolutionary mechanism for adding new amino acid sequence [39]. PolyQ stretches are also functionally associated to human disorders and it has been shown that the polyQ stretch length is correlated to the appearance of polyQ peptides in inclusion of highly insoluble aggregates in the nucleus or cytoplasm [for a review see [40]]. Thus, aggregation can compromise function by sequestering the protein away from their usual localization, but also because these aggregates are able to interact with several transcription factors harbouring short glutamine repeats [e.g. cAMP response element binding protein (CBP) and TATA-binding protein (TBP), [41,42]] via glutamine-glutamine interactions, or with other interaction partners [43]. Moreover, it has been shown that expanded polyQ tracts in genes involved in male sexual differentiation in tetrapods, result in abnormal sex differentiation. Breeding the Y chromosome in certain *Mus musculus domesticus *strains onto the inbred laboratory mouse strain, C57BL/6J (B6), results in hermaphroditic progeny, with different degrees of sex reversal, as a consequence of specific changes in the predicted polyQ amino acid sequence at this site [44]. Also, extended polyQ tracts in the transactivation domain of the androgen receptor (AR) are associated to increased risk of defective spermatogenesis and under masculinisation, depending on the genetic background [45]. Considering the above and although, the data available is rather small, it is tempting to speculate that the length of the glutamine stretch in DAX1 from fishes could be positively correlated to susceptibility to masculinisation by temperature. While in European sea bass, with a large number of polyQ, temperatures above 17°C (2–4°C above the natural spawning temperature) cause masculinisation [26], in *Onchorynchus *species (closely related to rainbow trout), zebrafish and tilapia (with a small number of polyQ), temperatures close to the lethal upper limit are required for some degree of masculinisation to occur [46-49]. In this hypothesis, the formation of DAX1 aggregates could limit the availability of active DAX1 protein and reduce its inhibitory action on masculinising factors, such as SOX-9 [16,17,50]. However, this does not explain the susceptibility to temperature in tetrapods, which appear to be highly dependent on kinetic properties of the aromatase enzyme [51].

The C-terminal of DAX1, presents a high degree of conservation in all species studied so far. To date, all mutations in adrenal hypoplasia congenita localize to the C-terminal part of the gene, suggesting that this region represents the critical domain for DAX1 function [52]. This domain has been shown to be responsible for transcriptional silencing activity in mammalian species in a similar way to a restricted subset of other members of the nuclear receptor super family: thyroid hormone (TR), the related oncogene product v-erbA, retinoic acid receptor (RAR), and the chicken ovalbumin upstream promoter transcription factor (COUP-TF) [11]. The silencing activity of DAX1 is possible due to a bipartite domain in the C-terminal [11] that corresponds to the helix 3 and helixes 11–12, according to the DAX1 protein structure prediction when aligning DAX1 with RAR, TR and v-erbA sequences [11,20]. The fact that all DAX1 sequences are highly conserved within these regions, suggests a common mechanism across the vertebrates. This is further supported by the presence of a highly conserved AF-2 core sequence at the C-terminal end.

In the Southern blot a single band was detected in males and females indicating that DAX1 is the product of a single gene and that it is not sex-specific. Moreover, genomic DNA amplification of the DAX1 gene in sea bass from both sexes has also shown that this gene has a two exon structure, separated by a single intron with 283 bp and conserved splice sites located at the same region of the ligand binding domain as all other DAX1 genes described so far. These results confirm what had been observed in the Southern blot analysis and thus emphasize that in this species the DAX1 gene is transcribed from a single gene. Northern blot analysis detected a ~1.3 kb transcript in gonads of mature (2-year old) fish of both sexes, similar to that isolated in the testis cDNA library. These results are similar to those obtained in tilapia [6], chicken [9] and alligator [7]. In contrast, no expression was detected in ovary or testis of adult frogs, which was suggested to be due to the fact that after metamorphosis is complete only spermatogonia could be found in testis, while in younger animals, Leydig cells could be detected [8]. The DAX1 mRNA was not confined to reproductive tissues but instead it was present in all tissues analysed. Although higher levels of DAX1 were apparent in brain, pituitary and gonads of female sea bass, the reverse was true for head kidney, kidney, digestive tract, liver, heart, gills and muscle. The same broad expression in tissues and differences in expression levels between male in female tissues have also been documented for tilapia, although male tilapia brain expressed higher levels of DAX1 [9]. In frog and mouse DAX1 is also expressed in several tissues other than the brain-pituitary-gonadal/adrenal axis [8,53]. ERα, another nuclear receptor, was also higher in female compared to male sea bass liver [54]. Nonetheless, the biological significance of this dimorphic pattern in non reproductive tissues is hard to interpret at present.

In the sea bass, DAX1 was detected as early as day 4 post hatching, well before the period of sex differentiation [21], in accordance with similar observations in other vertebrates [6,8,9,55], including zebrafish [38] and with its essential role in steroidogenic enzyme and interrenal/adrenal development [38,56,57]. In fact, recent observations in *Danio rerio *have underlined the role of DAX1 in the interrenal development where it has been found that there is a weak and transient expression of this gene at approximately 31 hours post fertilization (hpf) in the interrenal, and also that disruption of DAX1 function by morpholino oligonucleotides resulted in the down regulation of steroidogenic enzymes StAR and CYP11A in this tissue [38]. The expression of DAX1, however, did not follow a dimorphic expression pattern at any time during sea bass development, including the sex differentiation period. At 150 days (gonadogenesis) there was an apparent decrease in expression, although the tissue analysed was not exclusively gonads (body trunks). Coincidently, this is also the period of highest sensitivity to androgen treatments [58] and when there is a dimorphic pattern of androgen receptor (AR) α and 11β-hydroxylase mRNA expression with higher levels in the group dominated by males [59,60]. Considering this temporal pattern and also the documented regulation of DAX1 on AR in mammalian species, the DAX1 could be a candidate to regulate the effects of androgens on gonadal differentiation. In human, DAX1 protein is able to interact with the AR protein through direct interaction of the DAX1 N-terminal repeat domain with the AR C-terminal ligand binding and activation domain [17]. By blocking the interaction of the N-terminal and C-terminal of the AR, the DAX1 will also inhibit the ligand-dependent transcriptional activation of the AR, and thus influence the regulation of androgen- dependent gene transcription in the male reproductive system [17]. Despite the early expression of DAX1 in sea bass larvae, we could not detect any influence of temperature on DAX1 expression during the thermo sensitive period, possibly due to the fact that at this stage (4–60 dph) there is no gonadal development and thus, the expression levels obtained are from whole body larvae. In alligators, an increase in gonadal DAX1 expression was detected during the temperature- sensitive period, although, as found in sea bass, DAX1 expression was not sexually dimorphic during gonadogenesis [7].

Although no dimorphic pattern could be detected at the mRNA level during both thermosensitive period and gonadal differentiation period, it is possible that at the protein level, DAX1 is interacting or regulating other proteins that are involved in determining the sexes. Thus, future studies on protein interactions with the sbDAX1 protein should give us a better insight on the role of this protein in the process of gonadal differentiation. Also, immunocytochemistry or in situ hybridization should help to clarify the early pattern of gonadal DAX1 expression.

## Conclusion

In conclusion, we have isolated a full length cDNA from sea bass that has highest identity to other teleost fish DAX1. The mRNA expression of sbDAX1 shows a dimorphic pattern of expression as higher levels are detected in the female gonads, brain and pituitary than in males, although out of this axis this gene is substantially more expressed in tissues from males than from females. Sea bass DAX1 is expressed throughout ontogeny from day 4 post hatching until the end of gonadal differentiation (300 dph), although no dimorphic pattern is observed between males and females, suggesting that DAX1 is important for gonadal differentiation but is not sex specific. No effect of temperature was found in DAX1 mRNA expression throughout the temperature sensitive sex determining period.

## Competing interests

The author(s) declare that they have no competing interests.

## Authors' contributions

RSM cloned sbDAX1, did the phylogenetic analysis, analysed gene structure and expression and wrote the manuscript. LAMD cloned sbDAX1. CCM planned and carried out the experiments with larva. DMP participated in the discussion of results and wrote the manuscript. AVMC devised the study, participated in the planning of experiments, analysis and discussion of results, and wrote the manuscript. All authors read and approved the final manuscript.

## Supplementary Material

Additional File 1 Sea bass DAX1 full length nucleotide sequence and encoding protein. The 5'UTR is 116 bp long and ends with a partially conserved Kozak sequence (GCC ATG GCC), signalling the start codon. The open reading frame (ORF) is 906 bp long. Asterisc signals the stop codon of the ORF. The 3'UTR is 350 bp long, containing a polyadenylation signal (signalled in bold) and a poly A+ tail. Click here for file
