# Supplementary material for: Developmental expression of DAX1 in the European sea bass, Dicentrarchus labrax: lack of evidence for sexual dimorphism during sex differentiation
Source: Reprod Biol Endocrinol. 2007 May 30;5:19. doi: 10.1186/1477-7827-5-19 (PMC1891300; doi:10.1186/1477-7827-5-19)
Supplement: Additional File 1 — Sea bass DAX1 full length nucleotide sequence and encoding protein. The 5'UTR is 116 bp long and ends with a partially conserved Kozak sequence (GCC ATG GCC), signalling the start codon. The open reading frame (ORF) is 906 bp long. Asterisc signals the stop codon of the ORF. The 3'UTR is 350 bp long, containing a polyadenylation signal (signalled in bold) and a poly A+ tail. [file 1477-7827-5-19-S1.pdf]

```
GGCACGAGGGCTATCAGGGGCTAAGAAAACAGCGCTACCTCTGTCACTGACCTCCGGCGCTTCAAAGAGAC     71

                                                 M   A   T   L   E   G       6
AGCGGGACGCGCTGAGCTCCATCCCATGTAGAGCGGCCAGCGGCC ATG GCC ACG CTG GAG GGC      134

 C   R   C   R   G   A   S   G   R   N   N   N   S   I   L   Y   N   I      24
TGC CGC TGT CGG GGT GCC AGC GGC CGA AAC AAT AAC AGC ATC CTC TAC AAC ATT    188

 L   K   S   D   S   L   A   T   A   E   E   Q   Q   Q   Q   Q   Q   Q      42
TTG AAG AGT GAC AGC CTT GCG ACT GCC GAG GAG CAA CAA CAA CAA CAA CAA CAA    242

 Q   Q   Q   Q   Q   Q   H   P   Q   Q   Q   Q   T   L   Q   Q   L   F      60
CAA CAA CAA CAA CAA CAA CAT CCC CAA CAA CAA CAA ACC CTG CAA CAA TTG TTT    296

 H   K   T   S   S   T   A   A   P   A   S   L   Q   E   L   R   Q   Q      78
CAC AAG ACC TCC TCC ACC GCC GCC CCA GCC TCT CTG CAG GAG CTC CGG CAG CAG    350

 T   C   S   C   G   S   T   R   R   R   G   I   L   R   F   P   Q   V      96
ACT TGC TCC TGC GGC TCG ACC CGG CGC AGA GGT ATC CTC CGC TTC CCA CAG GTG    404

 T   C   K   A   A   S   A   V   L   V   K   T   L   R   F   V   K   N     114
ACG TGC AAA GCC GCC TCG GCG GTT CTG GTG AAG ACG CTG CGG TTC GTG AAA AAC    458

 V   P   C   F   R   E   L   P   E   D   D   Q   L   M   L   I   R   S     132
GTC CCC TGT TTT CGC GAG CTG CCG GAG GAC GAC CAG CTG ATG TTG ATC CGG AGC    512

 G   W   A   P   L   L   V   L   G   L   A   Q   D   R   V   D   F   E     150
GGC TGG GCA CCC TTG CTC GTG CTG GGA CTC GCA CAA GAC CGA GTG GAC TTT GAG    566

 T   T   E   T   V   E   P   S   M   L   Q   R   I   L   T   G   L   P     168
ACC ACG GAG ACC GTG GAG CCC AGC ATG CTG CAG CGC ATC CTC ACG GGT TTA CCA    620

 D   R   Q   S   E   V   P   A   G   Q   S   R   G   A   A   G   V   S     186
GAC AGG CAG AGC GAG GTG CCG GCT GGC CAA AGC AGG GGG GCA GCC GGG GTC TCT    674

 V   V   D   I   E   A   I   K   A   F   L   K   K   C   W   S   V   D     204
GTC GTG GAT ATC GAA GCT ATC AAA GCC TTC CTG AAG AAG TGC TGG AGT GTA GAT    728

 I   S   T   K   E   Y   A   Y   L   K   G   A   V   L   F   N   P   D     222
ATC AGT ACG AAG GAG TAT GCG TAC CTG AAA GGA GCT GTG CTG TTC AAC CCA GAT    782

 V   E   G   L   R   C   L   H   Y   I   Q   S   L   R   R   E   A   H     240
GTG GAG GGT TTG CGC TGC CTC CAC TAC ATC CAG TCT CTG CGT CGG GAA GCG CAC    836

 Q   A   L   N   E   H   V   R   L   I   H   R   E   D   T   T   R   F     258
CAG GCT CTG AAC GAG CAC GTC AGG CTG ATC CAC CGC GAG GAC ACG ACG CGG TTC    890

 A   K   L   L   I   A   L   S   M   L   R   A   I   N   P   L   V   V     276
GCC AAA CTG CTC ATA GCT CTG TCC ATG CTG AGG GCC ATC AAC CCG CTG GTG GTC    944

 A   Q   L   F   F   R   P   V   I   G   A   V   N   I   E   E   V   L     294
GCA CAA CTC TTC TTC AGA CCC GTT ATA GGG GCC GTC AAC ATC GAG GAG GTG CTC    998

 M   E   M   F   Y   G   K   *                                             312
ATG GAG ATG TTT TAC GGG AAA TAG GTC ACA GTC CAC AGT GCA CTG AGG TGA ATT   1052

CAG CAG ATT CAG ATG CCA GAT GGA CTG AAA TGC TCC AAT CTC TTA TTG AGT ATG   1106
AGA TGT GGG AAG ATG AAG AGG ATA ATA GGG ACT GAA TTT ACA ACT ACT GTG TGT   1160
TGG AGA TCT TCA AGG ACT GTG ACA GTG ACC AAC ACA GCC TAA GTG TGT GCG TGT   1214
GTG TAT GAG AGA ATG AGA AGA GAG AGT GTG TTG AGG GCA AAC TAT GTT ATA ATA   1268
GAG GAA AAA ACA AAG CAG CTG CTG GAA TTC ATA ACT TAT TTG TTT TTA TAT AAA   1322
CTA TTT TTC TAT AAT AAA GAA ATA CTC TCA GAA AAA AAA AAA AAA AAA AAC       1373
```
